# Neonatal treatment with scopolamine butylbromide prevents metabolic dysfunction in male rats

**DOI:** 10.1038/srep30745

**Published:** 2016-08-26

**Authors:** Ananda Malta, Aline Amenencia de Souza, Tatiane Aparecida Ribeiro, Flávio Andrade Francisco, Audrei Pavanello, Kelly Valério Prates, Laize Peron Tófolo, Rosiane Aparecida Miranda, Júlio Cezar de Oliveira, Isabela Peixoto Martins, Carina Previate, Rodrigo Mello Gomes, Claudinéia Conationi da Silva Franco, Maria Raquel Marçal Natali, Kesia Palma-Rigo, Paulo Cezar de Freitas Mathias

**Affiliations:** 1 Laboratory of Secretion Cell Biology, Department of Biotechnology, Genetics and Cell Biology, State University of Maringa, Maringá, PR, Brazil; 2 Health Sciences Institute, Federal University of Mato Grosso, Sinop, MT, Brazil; 3 Department of Morphophysiological Sciences, State University of Maringa, Maringá, PR, Brazil

## Abstract

We tested whether treatment with a cholinergic antagonist could reduce insulin levels in early postnatal life and attenuate metabolic dysfunctions induced by early overfeeding in adult male rats. Wistar rats raised in small litters (SLs, 3 pups/dam) and normal litters (NLs, 9 pups/dam) were used in models of early overfeeding and normal feeding, respectively. During the first 12 days of lactation, animals in the SL and NL groups received scopolamine butylbromide (B), while the controls received saline (S) injections. The drug treatment decreased insulin levels in pups from both groups, and as adults, these animals showed improvements in glucose tolerance, insulin sensitivity, vagus nerve activity, fat tissue accretion, insulinemia, leptinemia, body weight gain and food intake. Low glucose and cholinergic insulinotropic effects were observed in pancreatic islets from both groups. Low protein expression was observed for the muscarinic M_3_ acetylcholine receptor subtype (M_3_mAChR), although M_2_mAChR subtype expression was increased in SL-B islets. In addition, beta-cell density was reduced in drug-treated rats. These results indicate that early postnatal scopolamine butylbromide treatment inhibits early overfeeding-induced metabolic dysfunctions in adult rats, which might be caused by insulin decreases during lactation, associated with reduced parasympathetic activity and expression of M_3_mAChR in pancreatic islets.

The number of individuals who suffer from metabolic diseases is increasing worldwide in both wealthy and poor countries^1^. Although high caloric intake, sedentary behavior and genetics are the main risk factors for these diseases, stressful conditions during perinatal life, such as nutritional, metabolic and hormonal alterations, also contribute to the development of metabolic diseases in adult life. These observations support the Barker theory and the idea of metabolic programming, i.e., the developmental origins of health and disease concept, which is that early-life conditions can affect later health and development^2^,^3^,^4^,^5^.

Insulin and other hormones, such as leptin, are peripheral regulators of food intake and body adiposity; furthermore, insulin plays a key role in producing the neural connections that regulate feeding and glucose metabolism in adulthood. The central nervous system (CNS) and autonomic nervous system (ANS) govern important components of metabolism. In humans, the end of pregnancy is a crucial phase for developing neuronal connections^6^; however, in rodents, CNS development extends further, into the beginning of lactation^7^.

Insulin secretion is modulated by glucose and several neurotransmitters released from the peripheral autonomic nerves. The major neurotransmitter of the peripheral parasympathetic nervous system (PNS), i.e., acetylcholine (ACh), is known to facilitate the release of insulin in a glucose-dependent manner. This activity has been shown to be mediated by the activation of muscarinic acetylcholine receptors (mAChRs) located on the plasma membrane of pancreatic beta-cells^8^,^9^. ANS imbalances, including high parasympathetic and low sympathetic activity, are frequently observed in human and rodent models of overweight and obesity. In addition, these obese individuals exhibit high fasting insulinemia and insulin resistance^10^,^11^,^12^. Indeed, ANS imbalances have been suggested to be one cause of pancreatic beta-cell dysfunction^13^.

Neuroendocrine disruptions are induced by programming events during rat perinatal life, when the CNS is developing. For example, elevated insulin concentrations during critical periods of development might lead to malprogramming of the hypothalamic networks that regulate body weight and metabolism. In babies born to mothers with gestational diabetes, hyperinsulinemia has been shown to have a high correlation with the development of obesity during later life^14^. Similarly, transient hyperinsulinism during lactation led to rats becoming overweight and having an increased susceptibility to diabetes in adult life^15^.

Early hyperinsulinemia also occurs as a result of postnatal overnutrition and can lead to the development of permanent insulin resistance, obesity and diabetes^16^. In rodents, early postnatal overfeeding can be induced by reducing litter size^17^. Small litter (SL) rats present with early hyperinsulinemia; develop hyperphagia, high parasympathetic activity^18^ and impaired glucose tolerance; and become overweight in adulthood more than do normal litter (NL) rats^19^. Interestingly, early hypoinsulinemia observed in postnatally undernourished rats is associated with low vagus activity and a lean phenotype in later life^20^,^21^. In addition, suckling rats whose mothers were exposed to protein restriction during lactation exhibited low insulin levels and low body weights in adulthood^22^. In addition to its influence on metabolic homeostasis control via the brain and peripheral nervous system during perinatal life, insulin appears to play an important role in metabolic programming^23^,^24^.

It has been shown that an intravenous infusion of scopolamine butylbromide, also known as Buscopan, may attenuate the release of insulin in healthy men by competing with ACh for the M_3_mAChR subtype^25^,^26^. Binding M_3_mAChRs using muscarinic antagonists also inhibits insulin release in rodents^25^.

Therefore, in the current study, we hypothesized that decreasing insulin levels during lactation could attenuate later metabolic dysfunction induced by early postnatal overfeeding. We administered Buscopan during early lactation to decrease insulin levels and assess the effects on overfeeding-induced metabolism malfunction onset in adult SL rats.

## Results

### Insulin plasma levels throughout the suckling phase

**Figure 1:**
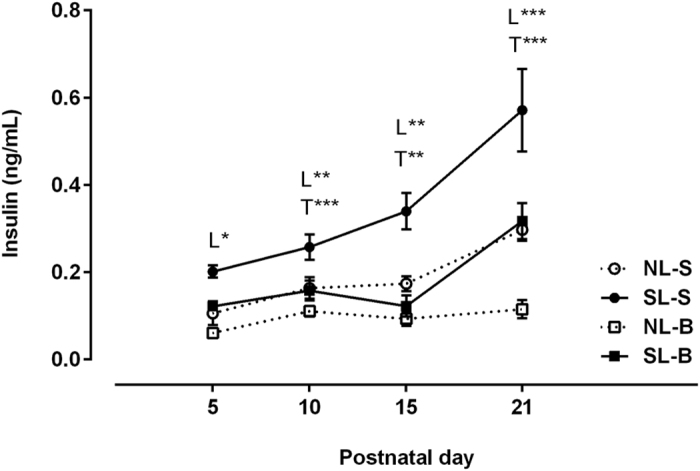
Effect of scopolamine butylbromide on offspring insulinemia throughout the suckling phase. NL-S, normal litter rats treated with saline solution; SL-S, small litter rats treated with saline solution; NL-B, normal litter rats treated with scopolamine butylbromide; SL-B, small litter rats treated with scopolamine butylbromide. L, litter size factor; T, treatment factor. *P < 0.05, **P < 0.01, ***P < 0.001, based on a two-way analysis of variance of 9–12 rats from each experimental group.

During the lactation period, the blood insulin levels of SL rats treated with saline (SL-S) were higher than their NL-S counterparts (*P*_L_ < 0.05), as shown in Fig. 1. The scopolamine butylbromide (B) treatment decreased the insulin levels of NL-B and SL-B rats (*P*_T_ < 0.01, Fig. 1).

### Scopolamine butylbromide treatment effects on biometrics, biochemical parameters and insulin sensitivity

As shown in Table 1, reducing litter size caused increases in the body weight (bw) of SL-S rats at weaning (21 days old) and in adulthood (90 days old) of 38% and 10%, respectively (*P* < 0.05). However, NL-B and SL-B rats showed 20% and 16% reductions in bw at weaning, respectively (*P* < 0.05), and a 10% reduction in bw at adulthood (both *P* < 0.05). Food intake increased by 5% in SL-S rats compared with that of NL-S rats (*P* < 0.05), while NL-B and SL-B rats showed a 10% reduction in food consumption (*P* < 0.05). Animals in the SL-S group showed weight increases of approximately 20% in all three pads (retroperitoneal, periepididymal and mesenteric) compared with that of animals in the NL-S group (*P*_L_ < 0.05). The drug treatment induced approximately 15% and 20% decreases in fats pads weight in NL-B and SL-B rats, respectively (*P*_T_ < 0.05). Leptin blood concentration was doubled in SL-S rats compared with that in NL-S rats (*P*_L_ < 0.01), and the level of leptinemia was decreased in NL-B and SL-B rats (*P*_T_ < 0.01). Fasting insulinemia was increased by 71% in SL-S rats compared with NL-S rats; in contrast, NL-B and SL-B rats showed decreases in fasting insulinemia of 42% and 70%, respectively, indicating an interaction (*P*_I_ < 0.05). SL-S rats presented an 11% increase in fasting glycemia compared with that of NL-S rats; while NL-B rats exhibited an 8% increase in glycemia compared with that of NL-S rats (*P* < 0.05), no effect was observed in SL-B animals. The rate constant for disappearance of plasma glucose (K_itt_) was decreased by 28% in SL-S rats compared with NL-S rats (*P* < 0.05). Although no significant difference was detected in NL-B rats, SL-B rats exhibited a 40% increase in K_itt_. The homeostasis model assessment of insulin resistance (HOMA-IR) was increased by 88% in SL-S rats compared with NL-S rats (*P* < 0.05). Although no significant difference was detected between NL-S and NL-B rats, SL-B animals exhibited a 68% decrease in HOMA-IR compared with that of SL-S rats, *P* < 0.05.

### Plasma glycemia and insulinemia during the intravenous glucose tolerance test (ivGTT)

**Figure 2:**
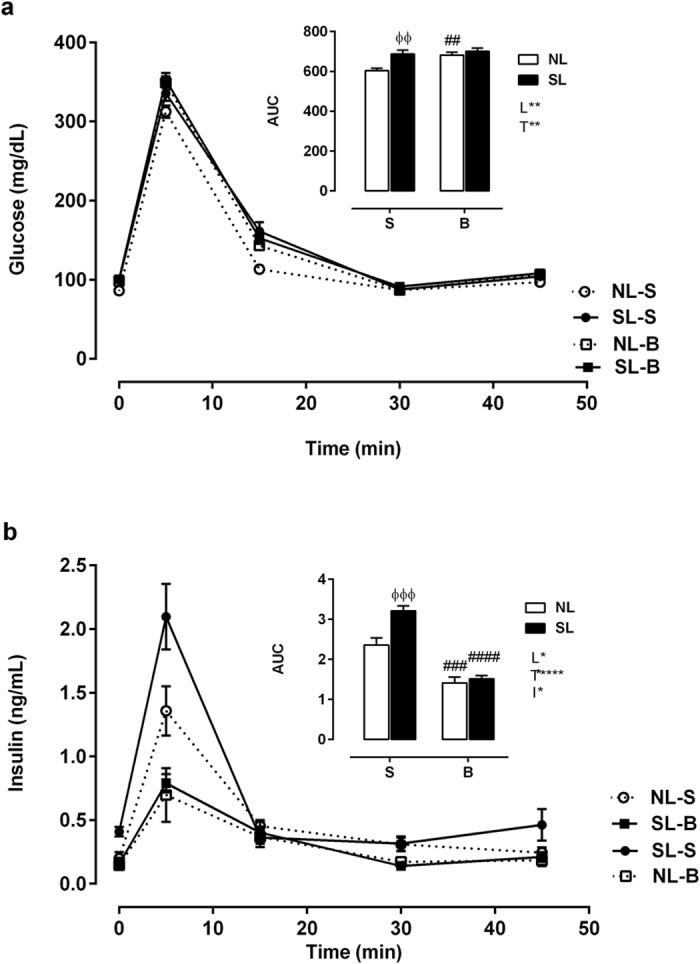
Blood glucose (**a**) and insulin (**b**) levels during the ivGTT. The upper panel of each figure represents the area under the curve (AUC). NL-S, normal litter rats treated with saline solution; SL-S, small litter rats treated with saline solution; NL-B, normal litter rats treated with scopolamine butylbromide; SL-B, small litter rats treated with scopolamine butylbromide. Data are expressed as the mean ± SEM of 9–17 rats from each experimental group. L, litter size factor; T, treatment factor; and I, interaction between litter size and treatment factors. *P < 0.05, **P < 0.01, ****P < 0.0001, based on a two-way analysis of variance. ^ɸɸ^*P* < 0.01 and ^ɸɸɸ^*P* < 0.001, statistical significance of differences between NL and SL; ^##^*P* < 0.01, ^###^*P* < 0.001 and ^####^*P* < 0.0001, statistical significance of saline versus scopolamine butyl bromide animals, based on the Tukey multiple comparisons test.

During the ivGTT, glycemia (Fig. 2a) and insulinemia (Fig. 2b) were increased by 13% and 36%, respectively, in SL-S rats compared with NL-S rats (*P* < 0.001), as indicated by the area under the curve (AUC) values. Although early drug treatment affected glycemia in the NL-B group (Fig. 2a), insulinemia was reduced by 40% and 50% in the NL-B and SL-B groups, respectively (*P* < 0.001).

### Pancreatic islet function and insulin secretion

**Figure 3:**
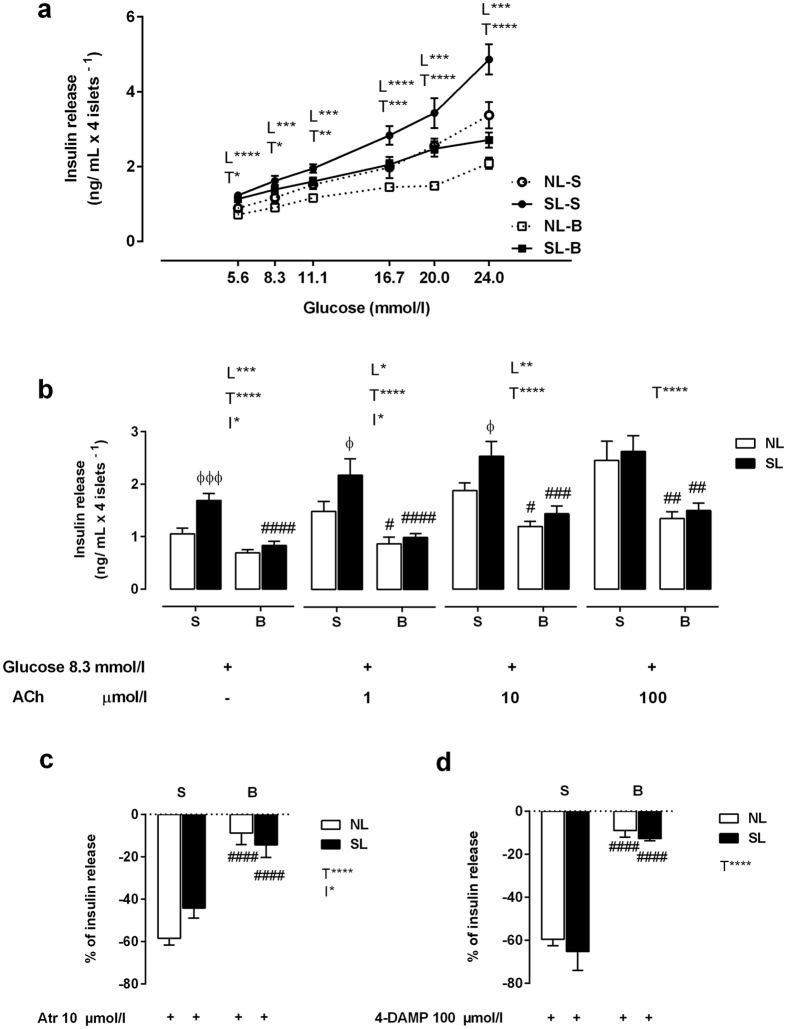
Pancreatic islet insulin secretion. Insulin secretion stimulated by different glucose concentrations **(a)**. Insulin secretion stimulated by 8.3 mmol/l glucose and potentiated by 1, 10 or 100 μmol/l ACh **(b)**. Inhibition of ACh insulinotropic effect (8.3 mmol/l + 10 μmol/l ACh in the presence of 10 μmol/l neostigmine) by the non-selective antagonist Atr (10 μmol/l) **(c)** and the antagonist selective to M_3_mAChR, 4-DAMP (100 μmol/l) **(d).** The results are presented as a percentage of insulin secretion. The line at 0 (**c**,**d**) represents 100% ACh insulinotropic action. The presence ( + ) and absence (–) of glucose, ACh, Atr and 4-DAMP is indicated below each corresponding bar. The data were obtained from the pancreatic islets of 6 rats from three different litters of each experimental group. NL-S, normal litter rats treated with saline solution; SL-S, small litter rats treated with saline solution; NL-B, normal litter rats treated with scopolamine butylbromide; SL-B, small litter rats treated with scopolamine butylbromide. L, litter size factor; T, treatment factor; and I, interaction between litter size and treatment factors. *P < 0.05, **P < 0.01, ***P < 0.001, ****P < 0.0001, based on a two-way analysis of variance. ^ɸ^*P* < 0.05 and ^ɸɸɸɸ^*P* < 0.0001, statistical significance of the differences between NL and SL; ^##^*P* < 0.01 and ^####^*P* < 0.0001, statistical significance of saline versus scopolamine butylbromide animals, based on the Tukey multiple comparisons test.

In all groups, pancreatic islets showed a dose-dependent increase in insulin secretion in response to glucose stimulation (Fig. 3a). Islets from SL-S rats secreted more insulin than islets from NL-S rats when stimulated by all the tested glucose concentrations (*P*_*L*_ < 0.001). The drug treatment reduced the glucose-induced insulin secretion of islets from NL-B and SL-B animals (*P*_*T*_ < 0.05). In addition, islets from rats in all groups showed dose-dependent increases in cholinergic response (Fig. 3b). However, incubation with increasing ACh concentrations showed that SL-S, SL-B and NL-B islets were less responsive to cholinergic action at all ACh concentrations than were NL-S islets (*P* < 0.05, Fig. 3b). Atropine (Atr), a nonselective muscarinic antagonist, inhibited the cholinergic response of islets isolated from all groups (*P* < 0.0001); however, this effect was reduced in NL-B and SL-B islets (Fig. 3c). Additionally, a muscarinic antagonist selective to the M_3_ subtype, 4-diphenylacetoxy-N-methylpiperidine methiodide (4-DAMP), decreased insulin secretion in islets from NL-S and SL-S rats; however, NL-B and SL-B islets were inhibited to a lesser extent (*P* < 0.0001, Fig. 3d).

### M_3_mAChR and M_2_mAChR protein expression in pancreatic islets

**Figure 4:**
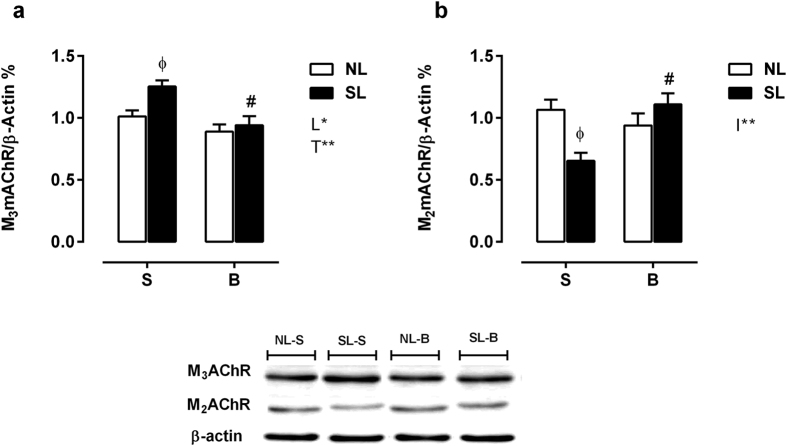
Measurement of M_3_ (**a**) and M_2_ (**b**) muscarinic receptor subtype protein levels in pancreatic islets. NL-S, normal litter rats treated with saline solution; SL-S, small litter rats treated with saline solution; NL-B, normal litter rats treated with scopolamine butylbromide treatment; SL-B, small litter rats treated with scopolamine butylbromide treatment. L, litter size factor; T, treatment factor; and I, interaction between litter size and treatment factors. *P < 0.05, **P < 0.01, based on a two-way analysis of variance. ^ɸ^*P* < 0.05, statistical significance of the differences between NL and SL; ^#^*P* < 0.05, statistical significance of saline versus scopolamine butylbromide animals, based on the Tukey multiple comparisons test (*n* = 3 different experiments using 3–4 rats from each experimental group).

M_3_mAChR expression was 23% higher in SL-S islets than in NL-S islets (*P* < 0.05), and SL-B islets presented a 24% reduction in M_3_mAChR expression compared with that of SL-S islets, *P* < 0.05; however, no differences were observed between NL-B and NL-S islets (Fig. 4a). Compared with NL-S islets, SL-S islets showed a 38% reduction in M_2_mAChR protein expression (*P* < 0.05). M_2_mAChR expression was increased in SL-B islets by 69% compared with that in SL-S islets (*P* < 0.05), while no changes were observed in NL-B islets (Fig. 4b).

### ANS activity

**Figure 5:**
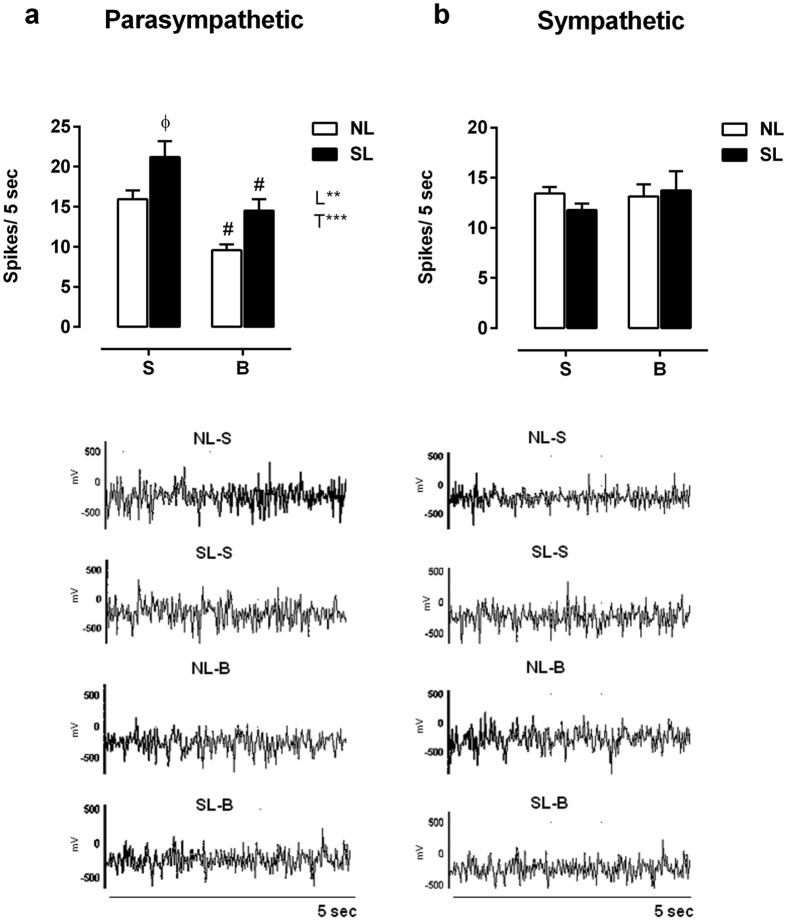
Parasympathetic (**a**) and sympathetic (**b**) electrical nerve activity. Data are expressed as the mean ± SEM of 15–17 rats from each experimental group. Lower panel graphs are representative records of nerve discharges of each group. NL-S, normal litter rats treated with saline solution; SL-S, small litter rats treated with saline solution; NL-B, normal litter rats treated with scopolamine butylbromide; SL-B, small litter rats treated with scopolamine butylbromide. L, litter size factor; and T, treatment factor. **P < 0.01, ***P < 0.001, based on a two-way analysis of variance. ^ɸ^*P* < 0.05, statistical significance of the differences between NL and SL; ^#^*P* < 0.05, statistical significance of saline versus scopolamine butylbromide animals, based on the Tukey multiple comparisons test.

The SL-S animals showed a 32% augmentation in vagus nerve electrical activity when compared with NL-S rats (*P* < 0.05), while the NL-B and SL-B groups showed reduced vagus nerve activity (*P*_T_ < 0.05, Fig. 5a). There were no changes in sympathetic electrical nerve activity in any group (Fig. 5b).

### Pancreatic morphology

**Figure 6:**
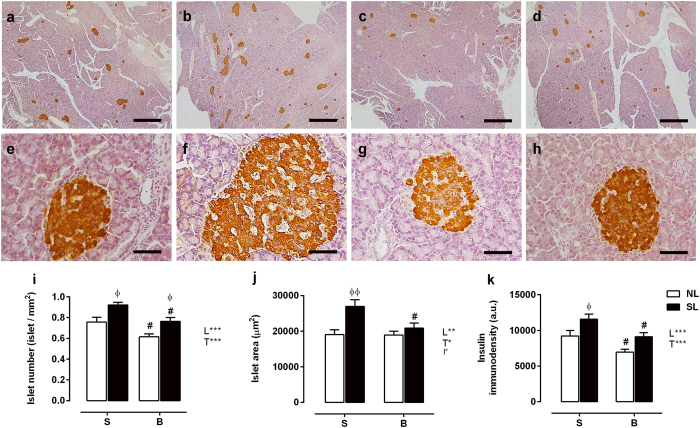
Effect of scopolamine butylbromide on histological and morphometric analysis of the pancreas. Representative images **(a**,**d**) × 20 magnification, scale bars = 1000 μm; (**e**,**h)** × 400 magnification, scale bars = 50 μm) of pancreatic sections immunostained with an anti-insulin antibody. NL-S, normal litter rats treated with saline solution **(a**,**e)**; SL-S, small litter rats treated with saline solution **(b**,**f)**; NL-B, normal litter rats treated with scopolamine butylbromide **(c**,**g)**; SL-B, small litter rats treated with scopolamine butylbromide **(d**,**h)**. A quantitative analysis of islet number, area and insulin immunodensity is shown in **(i–k)**, respectively. Data are presented as the mean ± SEM, obtained from 4 rats in each experimental group. A total of 160 islets were analyzed per group. L, litter size factor; T, treatment factor; and I, interaction between litter size and treatment factors. *P < 0.05, **P < 0.01 and ***P < 0.001, based on a two-way analysis of variance. ^ɸ^*P* < 0.05 and ^ɸɸ^*P* < 0.01, statistical significance of the differences between NL and SL; ^#^*P* < 0.05, statistical significance of saline versus scopolamine butylbromide animals, based on the Tukey multiple comparisons test.

Figure 6 shows the immunohistochemistry staining results. Pancreatic islet number, area and insulin immunodensity were significantly increased by 22%, 41% and 25%, respectively, in the SL-S group (*P* < 0.05) compared with the NL-S group (Fig. 6i–k). In contrast, the drug treatment induced an 18% decrease (*P* < 0.01) in islet number and a 25% decrease (*P* < 0.05) in insulin immunodensity in the NL-B group compared with the NL-S group. There were also morphometric reductions of 17%, 20% and 21% in islet number, area and insulin immunodensity (*P* < 0.05) in the SL-B group compared with the SL-S group.

## Discussion

The current study shows for the first time that a short scopolamine butylbromide treatment at the beginning of lactation is capable of preventing early overfeeding-induced metabolic dysfunction. These findings suggest that non-physiological insulin levels during lactation could be one cause of metabolic control disruption. In addition, the drug intervention induced a lean phenotype in rats from normal litters. Early treatment with scopolamine butylbromide induced a drop in insulin levels during the lactation period in both overfed and normal pups. An excess of insulin during the critical period of early development has been shown to change the function and structure of the hypothalamus. Indeed, inducing temporary hyperinsulinism at the beginning of lactation has been shown to lead to permanent metabolic alterations associated with disorganization in the hypothalamic nucleus, such as overweight, glucose intolerance and elevated blood pressure^26^. The most neural connections in primates are made at the end of pregnancy, while brain development continues throughout the lactation phase in rodents. Similarly to the brain, the pancreas initiates embryogenesis early, in the first trimester of pregnancy in primates^24^. However, in rodents, the pancreas begins to develop in mid-pregnancy, and islet cell proliferation and growth continue during the lactation phase^27^,^28^, which may indicate that decreasing insulin levels by using an insulin secretion inhibitor may modify normal beta-cell function. Insulin may also directly influence the programming of peripheral tissues, such as pancreatic tissue. In addition, insulin has been shown to contribute to the modulation of beta-cell function in inducing cell proliferation^29^.

As shown in the current work, low parasympathetic activity may also contribute to the deficit in beta-cell proliferation. Pancreatic islet quantity and volume were reduced in the NL-B and SL-B groups, both of which could be caused by low vagal activity in beta-cells^30^. Indeed, the scopolamine butylbromide-treated rats showed low insulin levels when fasting. The idea that the perinatal inhibition of insulin secretion can program adult offspring to develop hypoinsulinemia is reinforced by the fact that islets isolated from those animals responded poorly to glucose and ACh, which can be caused, at least partially, by vagal hypotonia. Interestingly, protein restriction during lactation induced a lean phenotype in normal adult rats; coincidently, those animals were hypoinsulinemic and exhibited low insulinotropic responses to glucose and ACh^20^. As expected, untreated SL rats, who were predisposed to metabolic dysfunction, had high parasympathetic activities, increased islet numbers and sizes, and high beta-cell densities; these findings are in agreement with data from the literature and from other obesity models^18^,^31^. High parasympathetic activity is associated with high glucose-induced insulin secretion and beta-cell proliferation in adult rats^13^, while vagotomy reduces glucose-induced insulin secretion and beta-cell proliferation^30^.

Low vagal activity may compromise intestinal motility, which can alter enteric hormone release. This process may contribute to the reduced insulinotropic response of beta-cells; however, the evidence of the vagal control of incretin hormone release is controversial^32^,^33^.

Scopolamine butylbromide is an anticholinergic drug that blocks mAChRs (via a high affinity for M_3_mAChRs); among other effects, this inhibits smooth muscle contraction and mitigates abdominal pain, such as pain in the uterus, intestines, kidneys, urinary bladder, and other tissues^34^. Many tissues respond to cholinergic stimuli^35^, and cells from these tissues, including pancreatic beta-cells, have muscarinic receptors that transduce cholinergic signals. Cholinergic stimulation via the PNS is pivotal to control oscillating insulin levels in the blood and maintain constant glycemia. ACh acts simultaneously with many other secretagogues, such as glucose, amino acids, metabolites, blood fluxes, temperature, and catecholamines, among others, which allows insulin levels to rise and fall depending on metabolic demands^36^.

The pancreatic beta-cell membrane expresses four subtypes of the mAChR: M_1_, M_2_, M_3_ and M_4_^37^. The odd mAChR subtypes are G protein-coupled receptors responsible for the cholinergic insulinotropic response, while the even mAChR subtypes attenuate the insulinotropic effects of the odd receptor subtypes. The M_3_mAChR is mostly recruited during cholinergic stimulation in beta-cells^8^. The current study shows that M_3_mAChR protein expression in pancreatic islets is reduced when SL animals are treated with scopolamine butylbromide in early life. We suggest that this drug, by unknown mechanisms, programs beta-cells to express low M_3_mAChR levels with low insulin secretion. In addition, islets from SL animals showed low M_2_mAChR protein expression; however, when these SL animals were treated with the antimuscarinic drug, M_2_mAChR protein expression increased to a level close to that observed in untreated NL rats. Upon binding ACh, the M_2_mAChR blocks its own insulinotropic effect, which controls cholinergic responses in pancreatic beta-cells^38^ and in other cells^39^. These results reinforce the role of muscarinic receptors in the insulin secretion activity of pancreatic beta-cells. Interestingly, knockout mice lacking the M_3_mAChR either throughout the whole body or specifically in beta-cells present a lean phenotype. In addition, these transgenic animals are hypophagic and hypoinsulinemic, and their pancreatic islets respond poorly to glucose and ACh^8^,^40^. Those hallmarks are similar to those found in the adult rat offspring that were perinatally treated with the antimuscarinic drug.

A lean phenotype can also be obtained by restricting protein during the first two-thirds of the lactation phase. These adult rat offspring also exhibit hypophagia, hypoinsulinemia, low body weight, low insulin secretion and low M_3_mAChR protein expression^41^. These findings support the observation that dietary restrictions during the first 14 days of lactation caused decreased insulin levels.

Both infants and adults have been treated with scopolamine butylbromide to alleviate pain. As a quaternary ammonium derivative, this drug does not appear to affect the CNS because it does not cross the blood-brain barrier (BBB)^34^. The BBB has been shown to not have completely matured by the early postnatal period in rodents^42^. However, mechanisms linked to peripheral changes, such as low insulin concentration, which also affects brain function, may be considered. Furthermore, we do not know whether scopolamine butylbromide can cross the BBB in the conditions of this study.

Considering that scopolamine butylbromide may affect other tissues, which eventually could contribute to metabolic programming, pancreatic beta-cells must be a distinct step in the mechanisms of metabolic dysfunctions. In particular, mAChRs could play an important role in the programming of a lean phenotype.

Independent of the programming mechanisms, it is important to consider that fluctuations in insulin concentration during perinatal life, including lactation, are a defining factor of later health and metabolic diseases.

## Materials and Methods

### Ethical approval

All animal procedures were performed according to the standards established by the Brazilian Association for Animal Experimentation (COBEA) and were approved by the Ethics Committee in Animal Research of the State University of Maringa (9117110914).

### Animal model and experimental design

Sets of 3 adult female Wistar rats and 1 male were mated. After 2 weeks, the pregnant females were separated and placed in individual cages with free access to water and food. At birth, all litters were adjusted to 9 pups for each dam (preferentially male). To induce early overfeeding, some litters were adjusted to 3 male pups per dam at the third day after birth, i.e., the SL group^16^,^43^,^44^. Litters containing 9 pups per dam served as controls, i.e., the NL group. Treatment with scopolamine butylbromide (Buscopan^®^, Boehringer) occurred in both groups from the first to the twelfth day of life via an intraperitoneal injection at a dose of 0.5 mg/kg bw/day, which was diluted with saline solution (i.e., the NL-B and SL-B groups). The dose was determined by the dose recommendation for the treatment of children less than 12 years old^34^. Other rats from both groups received a 0.9% saline solution in the same volume and period (i.e., the NL-S and SL-S groups). The offspring groups were weaned at day 21. We used five litters for each experimental group. The animals received water and a commercial diet (Nuvital^®^; Curitiba/PR, Brazil) *ad libitum*. During all phases of the protocol, the animals were placed in an environmentally controlled room (23 ± 3 °C; 12-hour light/dark cycle, from 07:00–19:00 h).

### Body weight, food intake and fat pad weight evaluation

Body weight (bw) was recorded at weaning or at 90 days of age. Food intake was determined every 2 days from weaning until 90 days of age and quantified by weighing the remaining food, subtracting that amount from the ration offered to the animals the day before, and dividing by the number of animals in the cage^45^. Then, AUC was calculated. At 90 days of life, the rats were anesthetized with thiopental (45 mg/kg bw) and decapitated. Fat pad stores (retroperitoneal, periepididymal and mesenteric) were removed and weighed to assess the state of obesity.

### Assessment of insulin plasma levels throughout the suckling phase

Blood samples of offspring from both groups that had fasted overnight were taken from the jugular vein to determine insulin concentrations on days 5, 10, 15 and 21 of life.

### ivGTT

A batch of 90-day-old rats underwent a surgical procedure for the ivGTT, as previously described^20^. Blood samples collected before (0 min) the ivGTT were used to assess fasting glycemia, insulinemia and leptinemia. The glucose and insulin responses during the GTT were calculated by estimating the total area under the glucose curve using the trapezoidal method^46^. The HOMA-IR was used as a physiological index of insulin resistance. This index was determined from fasting glucose and fasting insulin concentrations using the following formula: HOMA-IR = (fasting insulin [ng/ml] × fasting glucose [mg/dl])/22.5)^47^.

### Intraperitoneal insulin tolerance test

Another group of rats that had cannulas implanted, as described above, were fasted for 6 h prior to an intraperitoneal insulin tolerance test (1 U/kg bw), performed as previously reported^48^. Thereafter, the K_itt_ was calculated as previously described^49^.

### Pancreatic islet isolation and insulin secretion stimulation

Pancreatic islets were isolated by collagenase digestion and washed with Hank’s solution, as previously described^50^. The islets (four islets per well) were preincubated for 60 min at 37 °C in Krebs solution containing 5.6 mmol/l glucose in a mixture of 95% O_2_: 5% CO_2_ at pH 7.4 to stabilize insulin secretion. To study the responses to the insulinotropic effects of different glucose concentrations, after the preincubation, the batch of islets was incubated for an additional 60 min under the experimental conditions with 5.6, 8.3, 11.1, 16.7, 20.0 or 24.0 mmol/l glucose. To study mAChRs function, following preincubation (glucose, 5.6 mmol/l), another batch of islets was incubated for an additional 60 minutes in Krebs solution containing either 8.3 mmol/l glucose or 8.3 mmol/l glucose plus 1, 10 or 100 μmol/l ACh in the presence of 10 μmol/l neostigmine to block acetylcholinesterase action in the islets. To block mAChRs function, one of the two following cholinergic antagonists was added to Krebs-Ringer solution containing 8.3 mmol/l glucose + 10 μmol/l ACh in the presence of neostigmine 10 μmol/l: the non-selective mAChR antagonist Atr (10 μmol/l) or the selective mAChR subtype M3 antagonist, 4-DAMP (100 μmol/l)^41^.

### Blood glucose levels

Glucose concentrations was measured by the glucose oxidase method^51^ with a commercial kit (*Gold* Analisa^®^, Belo Horizonte, MG, Brazil).

### Insulin level analyses

Insulin concentration, in plasma samples and islet cultures, was determined by radioimmunoassay^52^. The insulin intra- and interassay variation coefficients were 9.8 and 12.2%, respectively.

### Leptin level quantification

At 90 days of life, leptin plasma levels were determined using an ELISA kit (Enzo^®^ Life Sciences, Plymouth Meeting, PA, USA). The leptin intra- and interassay variation coefficients were 5.9 and 7.2%, respectively.

### Western blotting analyses

The M_2_mAChR and M_3_mAChR protein contents in pancreatic islets isolated from 90-day-old rats were determined by immunoblotting. A pool of islets from each experimental group was collected, frozen, and subjected to maceration in Radio Immunoprecipitation Assay (RIPA) buffer (50 mmol/l Tris; 150 mmol/l NaCl; 1.0% Triton X-100; 0.1% SDS; 5 mmol/l EDTA; 50 mmol/l NaF; 30 mmol/l sodium pyrophosphate; 1 mmol/l sodium orthovanadate) containing a protease inhibitor cocktail (Roche^®^). The homogenate was centrifuged at 10000 rpm for 5 min at 4 °C. Total protein content was determined using a BCA^TM^ Protein Assay Kit (Thermo Scientific^®^, Rockford, IL, USA) and a microplate reader (Multi-Mode Reader, FlexStation^®^ 3 Benchtop, Molecular Devices, Sunnyvale, CA, USA). The samples were treated with Laemmli sample buffer (w/v: glycerol, 20%; β-mercaptoethanol, 10%; 10% sodium dodecyl sulfate (SDS), 40%; and 0.5 mol/l Tris at pH 6.8, 0.5%; plus deionized water and bromophenol blue)^53^.

Total protein extracts (30 μg) from the islets were separated by 12% SDS-PAGE at 150 V for 60 min. The proteins were then transferred from the gel to a polyvinylidene difluoride (PVDF) membrane by a Trans-Blot^®^ turbo system (Bio-Rad^®^ Laboratories, Hercules, CA, USA) and were then blocked with 5% BSA in Tween-Tris-buffered saline (TTBS; Tris-HCl, 1 mol/l; NaCl, 5 mol/l; and Tween 20, 0.05%, v/v) for 90 min under continuous shaking. The primary antibodies anti-M3AChR (1:1000) and anti-M2AChR (1:1000) were obtained from Sigma Aldrich. PVDF membranes were washed three times with Tween–TBS (0.1%) and were incubated for 1 h with the appropriate secondary antibodies conjugated to biotin (Santa Cruz Biotechnology, Inc). Then, the membranes were incubated with streptavidin-conjugated HRP (Caltag Laboratories, Burlingame, CA, USA). The immunoreactive proteins were visualized using an ECL Prime kit and Image Quant LAS (GE Healthcare, Buckingham, Shire, UK). The bands were quantified by densitometry using ImageJ 1.4 software (Wayne Rasband, National Institutes of Health, Bethesda, MA, USA). β-Actin protein content (Santa Cruz Biotechnology^®^, Santa Cruz, CA, USA) was utilized for data normalization^41^.

### Autonomic nerve activity assessment

At 90 days of life, after an overnight fast, a batch of rats from all of the experimental groups was anesthetized with thiopental (45 mg/kg bw). A longitudinal surgical incision was made in the anterior cervical region. The left vagus superior and sympathetic branch nerves were isolated from the superior cervical ganglia, and a pair of silver recording electrodes (0.6 mm in diameter) was placed underneath; the electrodes were then covered with silicone oil to prevent dehydration. The electrodes were connected to an electronic device (Bio-Amplificator; Insight Equipamentos, Ribeirão Preto, Brazil) that amplified the electrical signal prior to filtering frequencies lower than 1 kHz and higher than 80 kHz. The signal output was acquired using Insight software (Insight software) and stored on a computer. The animals were placed in a Faraday cage to avoid any electromagnetic interference during the data-acquisition period^54^,^55^.

### Immunostaining and morphometric analysis of the pancreas

Pancreatic samples from 90-day-old rat offspring were fixed in 10% dehydrated formalin, cleared and embedded in histological paraffin. The fixed samples were sectioned into semi-serial, 5-μm-thick slices using a microtome. After deparaffinization, the sections were rehydrated and blocked against endogenous peroxidase activity with 3% H_2_O_2_. The sections were then washed in 0.01 M phosphate-buffered saline (PBS, pH 7.4) and incubated with 10% non-immune goat serum (Histostain-Plus^®^, Invitrogen) for 10 min. After blocking, the sections were incubated with an anti-insulin monoclonal antibody diluted at 1:500 (Sigma^®^, St. Louis, MO, USA) for 1 h at room temperature. After washing with 0.01 M PBS, the sections were incubated with a specific biotinylated secondary antibody (Histostain-Plus^®^, Invitrogen) for 10 min. Then, the sections were incubated with the enzyme streptavidin-peroxidase conjugate (Histostain-Plus^®^, Invitrogen) for 10 min and washed twice for 5 min. The streptavidin–biotin complexes were detected with diaminobenzidine chromogen solution (Histostain-Plus^®^ Invitrogen). Counterstaining with hematoxylin was performed for 15 s.

The number of pancreatic islets in the pancreas (number of islets/cm^2^) was quantified by insulin-immunostained pancreatic cuts (2 cuts/animal) in a sectional area of 40 islets/animal/group. Images were obtained in TIFF format using an optical microscope with 2 × and 40 × objectives coupled with a QColor 3 camera (Olympus, Tokyo, Japan). Analyses were performed using Image Pro Plus^®^ version 4.5 software 56.

### Statistical analysis

The results are expressed as the mean and the standard error (SEM), and the results were subjected to a D’Agostino Pearson normality test to assess the Gaussian distribution. Data were submitted to variance analysis by two-way ANOVA. Differences between the means were evaluated by Tukey’s post hoc test. A *P* value < 0.05 was considered significant regarding the main effects of litter size (L), treatment (T), their interaction (I; litter size vs treatment) and the differences between groups. Tests were performed using GraphPad Prism version 6.0 for Windows (GraphPad Software, Inc. San Diego, CA, USA).

## Additional Information

**How to cite this article**: Malta, A. *et al.* Neonatal treatment with scopolamine butylbromide prevents metabolic dysfunction in male rats. *Sci. Rep.* **6**, 30745; doi: 10.1038/srep30745 (2016).

## Figures and Tables

**Table 1:** Effect of scopolamine butylbromide on biometric and biochemical parameters.

Parameters	NL-S	SL-S	NL-B	SL-B	Factors		
					L	T	I
Body weight at 21 days of age (g)	46.2 ± 1.13	63.81 ± 1.39	36.9 ± 0.81	53.42 ± 0.78	****	****	ns
Body weight at 90 days of age (g)	382.3 ± 6.8	421.8 ± 7.7	345.1 ± 10.1	383.5 ± 8.6	****	***	ns
Retroperitoneal fat pad (g/100 g bw)	1.34 ± 0.06	1.61 ± 0.07	1.12 ± 0.03	1.27 ± 0.05	***	****	ns
Periepididymal fat pad (g/100 g bw)	1.13 ± 0.04	1.30 ± 0.06	0.98 ± 0.01	1.00 ± 0.02	*	****	ns
Mesenteric fat pad (g/100 g bw)	0.79 ± 0.03	0.97 ± 0.06	0.65 ± 0.02	0.78 ± 0.02	***	****	ns
Food intake (g; AUC)	214.1 ± 2.49	236.6 ± 1.92	192.0 ± 4.8	214.3 ± 5.86	***	***	ns
Fasting glycemia (mg/dL)	90.3 ± 2.05	100.3 ± 3.9	98.4 ± 1.39	101.2 ± 0.58	**	*	ns
Fasting insulinemia (ng/mL)	0.21 ± 0.02	0.36 ± 0.03	0.12 ± 0.07	0.11 ± 0.06	ns	****	*
Fasting leptinemia (pg/mL)	421.5 ± 88.4	843.4 ± 140.7	120.7 ± 72.7	314.2 ± 165.5	**	**	ns
K_itt_ (%/min)	3.11 ± 0.34	2.22 ± 0.08	2.78 ± 0.19	3.11 ± 0.28	ns	ns	*
HOMA-IR	1.19 ± 0.22	2.24 ± 0.35	0.90 ± 0.13	0.71 ± 0.13	ns	***	*

All data are expressed as the mean ± SEM of 15–25 rats from 5 different litters. NL-S, normal litter rats treated with saline solution; SL-S, small litter rats treated with saline solution; NL-B, normal litter rats treated with scopolamine butylbromide; SL-B, small litter rats treated with scopolamine butylbromide. L, litter size factor; T, treatment factor; and I, interaction between litter and treatment factors. *P < 0.05, **P < 0.01, ***P < 0.001, ****P < 0.0001 and ns, no significant difference, based on a two-way analysis of variance.
